# Detection of Antibodies to Seven Priority Pathogens in Backyard Poultry in Trinidad, West Indies

**DOI:** 10.3390/vetsci5010011

**Published:** 2018-01-20

**Authors:** Arianne Brown Jordan, Pompei Bolfa, Silvia Marchi, Shakera Hemmings, Tashard Major, Rod Suepaul, Lemar Blake, Christopher Oura

**Affiliations:** 1 Department of Basic Veterinary Sciences, School of Veterinary Medicine, The University of the West Indies (St. Augustine), Eric Williams Medical Sciences Complex, Mount Hope, Trinidad and Tobago; arianne.brown@my.uwi.edu (A.B.J.); Rod.Suepaul@sta.uwi.edu (R.S.); Lemar.Blake@sta.uwi.edu (L.B.); 2 Department of Biomedical Sciences, Ross University School of Veterinary Medicine, Basseterre, Saint Kitts and Nevis; pbolfa@rossvet.edu.kn (P.B.); smarchi@rossvet.edu.kn (S.M.); 3 School of Veterinary Medicine, The University of the West Indies (St. Augustine), Eric Williams Medical Sciences Complex, Mount Hope, Trinidad and Tobago; shakera.hemmings@my.uwi.edu (S.H.); tashard.major@my.uwi.edu (T.M.)

**Keywords:** chickens, Trinidad and Tobago, Avian influenza, infectious bronchitis virus, infectious bursal disease virus, Newcastle disease virus, West Nile virus, *Salmonella* enteritidis, *Mycoplasma gallisepticum*, *Mycoplasma synoviae*

## Abstract

Backyard poultry farms in Trinidad and Tobago (T&T) play a vital role in providing food and income for rural communities. There is currently no information on the presence and circulation of pathogens in backyard poultry farms in T&T, and little is known in relation to the potential risks of spread of these pathogens to the commercial poultry sector. In order to address this, serum samples were collected from 41 chickens on five backyard farms taken from selected locations in Trinidad. Samples were tested for antibodies to seven priority pathogens of poultry by enzyme-linked immunosorbent assay (ELISA). Antibodies were detected in 65% (CI 95%: 50–78%) of the sampled birds for Infectious bronchitis virus (IBV), 67.5% (CI 95%: 52–80%) for Infectious bursal disease virus (IBDV), 10% (CI 95%: 4–23%) for Newcastle disease virus (NDV), 0% (CI 95%: 0–0%) for Avian influenza virus (AIV), 0% (CI 95%: 0–0%) for West Nile virus (WNV), 31.7% (CI 95%: 20–47%) for *Mycoplasm gallisepticum/synoviae* and 0% (CI 95%: 0–0%) for *Salmonella enterica* serotype Enteritidis. These results reveal the presence and circulation of important pathogens of poultry in selected backyard farms in Trinidad. The results provide important information which should be taken into consideration when assessing the risks of pathogen transmission between commercial and backyard poultry farms, as well as between poultry and wild birds.

## 1. Introduction 

Trinidad and Tobago (T&T) has a large and thriving commercial poultry industry and is self-sufficient in meat and egg production [1]. It is estimated that over 37 million chickens were slaughtered for consumption in T&T in 2016 [2]. The country also has a significant number of small backyard poultry farm operations on both islands, which historically has been a source of food as well as a hobby for owners [3]. Backyard farms are considered to be small holdings of poultry on family dwellings with approximately 3–100 birds. The products from backyard farms are commonly used for home consumption and for small scale selling within local communities. Backyard farmers often rear mixed poultry flocks along with other animal species. The birds are usually free range and are typically not vaccinated. In T&T, backyard poultry farms occasionally experience devastating morbidity and mortality events, which often go unreported and undiagnosed. 

Various pathogens have been suspected to cause disease in backyard poultry farms in T&T, but definitive diagnoses are seldom made. Poultry kept in backyard settings tend to have close interactions with wild birds, including waterfowl and migratory species, putting them on the frontline for exposure to pathogens like Avian influenza virus and Newcastle disease virus that are carried by such birds. Additionally, the lack of farm biosecurity on backyard farms makes the birds more susceptible to certain pathogens [4]. In this study, a serological approach was used to determine antibody levels for seven priority pathogens in backyard poultry, chosen based on their zoonotic, veterinary health and public health importance. Pathogens selected were Avian influenza virus (AIV), Infectious bronchitis virus (IBV), Infectious bursal disease virus (IBDV), Newcastle disease virus (NDV), West Nile virus (WNV), *Salmonella enterica* serotype enteritidis (SE) and *Mycoplasma gallisepticum/synoviae* (*M. gall/synoviae*).

The only previous study on backyard poultry in T&T was a poultry production survey conducted in Northern Trinidad that was published in 1949/1950. This study revealed that ‘peasant’ and backyard farms experienced confirmed cases of Fowl pox and an undiagnosed ‘mystery disease’ [3]. Although some work has been conducted into pathogens affecting poultry in backyard settings in the Caribbean, this is the first such study to take place in T&T for a wide range of pathogens. The objective of this study was to determine if the selected high priority pathogens of poultry were present and circulating in backyard poultry in Trinidad and to assess the risks that pathogen circulation in backyard poultry poses to the commercial poultry sector on the island.

## 2. Materials and Methods

Chicken samples were collected from January to April 2015 based on convenience and accessibility of backyard poultry farms in selected locations on the island of Trinidad. A total of 41 samples were collected from mature (>12 months) unvaccinated birds on five backyard farms. The sampled birds exhibited no visible signs of clinical disease at the time of sampling. Blood samples were collected in red top tubes and were transported on ice to the laboratory where the serum was separated and stored at −80 °C. Serum samples were tested by enzyme-linked immunosorbent assay (ELISA) for the presence of antibodies against the various pathogens using commercial kits and following manufacturer’s instructions. Test kits for AIV, IBV, IBDV, NDV and WNV were ID Screen brand kits (IDVet) while both SE and *M. gall/synoviae* bacterial kits were Idexx brand kits (Table 1). Sensitivity and specificity levels of 98% and a 95% confidence level were used to determine confidence intervals (Table 2).

## 3. Results

For the viral pathogens, antibodies against IBV, IBDV and NDV were detected at levels of 65% (CI 95%: 50–78%), 67.5% (CI 95%: 52–80%) and 10% (CI 95%: 04–23%) in the tested bird samples. No antibodies against AIV and WNV were detected in the birds. For the bacterial pathogens, antibodies against *M. synoviae* were detected at levels of 31.7% (CI 95%: 20–47%), whereas no antibodies were detected for SE. The results from the study are summarized in Table 2. The results indicate the presence and circulation of IBV, IBDV, NDV and *M. gall/synoviae* in backyard poultry on the sampled farms in Trinidad, however antibodies to WNV and SE were not identified to be present. 

## 4. Discussion

This report describes the presence/absence of antibodies to seven priority pathogens of chickens in a small selection of backyard farms in Trinidad. Due to the small sample size, it was not possible to interpret the lack of detection of antibodies to Avian Influenza virus, West Nile Virus and Salmonella enterica. The results do however reveal the presence of antibodies and likely circulation of four priority pathogens (IBV, IBDV, NDV and *M. gall/synoviae*) in backyard chickens in Trinidad. Further, more detailed studies are required in order to fully understand the prevalence of these pathogens in backyard chickens and the impact of these pathogens on the poultry industry in Trinidad as a whole. 

Backyard poultry are thought to be at higher risk than intensively produced poultry of contracting viruses like AIV, NDV and WNV due to their close contact with wild birds that can act as reservoirs of these viruses [5,6,7,8]. The open layout of backyard farms often results in wild birds interacting closely with poultry species. This level of interaction is even more significant when there are open water sources on or near farms, as is often the case. T&T is also along the winter migratory routes for many migratory bird species, including waterfowl. Many of these migratory birds have been implicated as carriers of viral pathogens such as AIV, NDV and WNV. One such bird is the blue winged teal, from which AIV and NDV were isolated in Barbados [9]. In this study no antibodies were detected in the sampled backyard birds against AIV. A recent study investigating seroprevalence levels for AIV in domestic layer birds also showed no evidence for circulation of AIV [10]. Continued surveillance of backyard poultry for the presence of AIV is however important as backyard birds are often in close contact with potentially infected wild birds, especially during the migration season.

Wild birds are also capable of carrying and transmitting NDV to poultry. The results of this study indicate that low levels of NDV antibodies (10%) were present in backyard poultry. This relatively low seroprevalence level in backyard poultry contrasts a recent study carried out in T&T in which up to 80% of unvaccinated layer birds sampled in Trinidad were antibody positive to NDV [10]. These results suggest that exposure to NDV is lower in backyard poultry compared to commercial layer poultry. Farm management practices, species and age of birds and exposure to infected birds/environments could have contributed to the observed difference in seroprevalence levels. As NDV is a highly infectious virus, more rapid spread would be expected between layer birds kept in close contact under intensive conditions, than between backyard poultry kept under more extensive conditions. Given that no clinical signs of disease were noted in the birds sampled in this study, it is likely that either lentogenic, or possibly attenuated vaccine strains of NDV may be circulating and infecting the birds. In order to elucidate this, it would be necessary to carry out molecular characterization of the circulating viruses.

WNV, which is also carried by wild birds and is transmitted through mosquito bites, has also been detected in the region in both wild birds and domestic poultry. WNV has been detected in many Caribbean countries including Puerto Rico, Cuba, Dominican Republic, Jamaica and Guadeloupe, soon after the virus was introduced into the Americas [11,12,13,14]. Guadeloupe has carried out several seroprevalence studies in the period 2002–2004, to look for the presence and spread of the virus. In these studies, antibodies to WNV were detected in backyard farms as well as on domestic chicken farms [15,16]. Surveillance activities carried out in Trinidad in 2004 found 2 domestic Muscovy duck to be seropositive for WNV [17,18], although no virus was isolated. In this current study, no antibodies to WNV were observed in the sampled backyard birds.

Although less associated with transmission from wild birds, IBV and IBDV are extremely important viruses affecting poultry worldwide, including in the Caribbean. A recent serological study revealed high levels of antibodies to both viruses in layer birds in T&T. Antibody detection levels of close to 100% were reported for both viruses on both islands of Trinidad and Tobago [10]. The lower prevalence levels for IBV (65%) and IBDV (68%) reported in this study in backyard birds compared to intensively farmed layer birds may again be a result of multiple factors as described above for NDV. Both IBV and IBDV are widely vaccinated against in commercial poultry production units in T&T, however many smaller layer enterprises, as well as backyard farms, do not vaccinate their birds. It is however unclear whether the IBV and IBDV strains circulating in backyard farms are local field strains or vaccine derived strains. Molecular characterization of the circulating strain is needed to answer this question.

*M. synoviae* and *M. gallisepticum* can cause symptomatic respiratory tract infections in poultry and are often associated with IBV and NDV infections [19]. More commonly however, avian mycoplasmosis presents itself asymptomatically which, though unseen, can cause immunosuppression and poor health in infected birds [20]. There have been no previous reports of *M. synoviae* or *M. gallisepticum* in poultry in T&T and there are few World Organisation for Animal Health (OIE) reports of its presence and detection in poultry in the Caribbean [21]. In this study, 31.7% of the backyard birds tested had antibodies to *M. gall/synoviae,* indicating that one or both of these bacterial species were present in birds on the sampled farms. A lower level of detection is expected in backyard compared to intensively kept poultry, as multiple investigations have reported that smaller less densely populated flocks have lower levels of infection than larger more densely populated commercial flocks [22]. Further work is needed to determine if the circulating strains of *Mycoplasma* in T&T are pathogenic, as a wide range of pathogenicity and virulence factors are associated with different isolates of *M. synoviae* [23,24]. 

The results of this study revealed no antibodies to be present for SE in the backyard poultry that were sampled. This was surprising given that *Salmonella* has been detected in poultry houses and pluck shops throughout Trinidad [25,26,27], though these detections have been through bacterial enrichment and culture. Salmonellosis is the primary cause of food borne illness in T&T and the wider Caribbean and is of great public health concern. SE is one of the most common *Salmonella* serotypes isolated from both human and animal sources (poultry, eggs and egg products) in T&T, however there are multiple other Salmonella serotypes also implicated in causing disease in animals and humans [28,29]. As with *Mycoplasma* species, farm size is an important factor affecting *Salmonella* prevalence on farms. Larger more intensive operations have been found to have higher levels of *Salmonella* infection and transmission rates than smaller farms and backyard type farms [26,30].

## 5. Conclusions

This study confirms the presence of IBV, NDV, IBDV and *M. gall/synoviae* in unvaccinated backyard poultry in T&T and provides no evidence for the circulation of WNV and SE in the birds. Due to the limited sample size, it is not possible to extend the conclusions of this small study to the entire backyard poultry population in T&T. Nevertheless, the results highlight the presence of four important pathogens, emphasizing the risks that these pathogens pose to the backyard and commercial poultry sectors in T&T. The results emphasize the need for changes in farm management and biosecurity practices in the backyard poultry sector, in order to reduce the exposure of birds to these pathogens. The selected use of vaccines in this sector may also be recommended. The study also underlines the need for continuous surveillance and molecular identification and monitoring of disease in the non-commercial backyard poultry sector, as backyard poultry may act as sentinels for the introduction of new pathogens into the country and region through wild birds. The backyard poultry sector should be strongly supported as it plays a highly significant role in contributing to sustainable food security practices in local communities across the Caribbean region.

## Figures and Tables

**Table 1:** ELISA kits used to detect antibodies against various backyard poultry pathogens.

Virus	Test kit used	Manufacturer
Avian Influenza virus	ID Screen® Influenza A Antibody Competition Multi-species	ID.Vet
Infectious bronchitis virus	ID Screen® Infectious Bronchitis Indirect	ID.Vet
Infectious bursal disease virus	ID Screen® IBD Indirect	ID.Vet
Newcastle disease virus	ID Screen® Newcastle Disease Indirect	ID.Vet
West Nile virus	ID Screen® West Nile Competition Multi-species	ID.Vet
*Salmonella* enteritidis	IDEXX SE Ab Test	Idexx
*Mycoplasma gallicepticum/ synoviae*	IDEXX MG/MS Ab Test	Idexx

**Table 2:** Antibody detection levels for 7 priority pathogens of backyard chickens in Trinidad, West Indies.

	Positive% (95% CL)
Avian Influenza virus	0% (0,0)
Infectious bronchitis virus	65% (50,78)
Infectious bursal disease virus	67.5% (52,80)
Newcastle disease virus	10% (04,23)
West Nile virus	0% (0,0)
*Salmonella* enteritidis	0% (0,0)
*Mycoplasma gallisepticum/ synoviae*	32% (20,47)

CL: Confidence limits.
